# Study the Effect of Conjugate Novel Ultra-Short Antimicrobial Peptide with Silver Nanoparticles against Methicillin Resistant *S. aureus* and ESBL *E. coli*

**DOI:** 10.3390/antibiotics11081024

**Published:** 2022-07-30

**Authors:** Rula M. Darwish, Ali H. Salama

**Affiliations:** 1 Department of Pharmaceutics and Pharmaceutical Technology, School of Pharmacy, The University of Jordan, Amman 11942, Jordan; 2 Department of Pharmacy, Faculty of Pharmacy, Middle East University, Amman 11831, Jordan; asalama@meu.edu.jo

**Keywords:** antibiotic resistance, silver nanoparticles, ultra-short antimicrobial peptide, nanosilver, antimicrobial peptides

## Abstract

*Background:* Bacterial resistance is a challenging limitation in infection treatment. This work evaluates the potential antibacterial activity of conjugation of Tryasine peptide with silver nanoparticles against selected pathogens. *Materials and Methods:* The peptide Tryasine was produced using three subunits of tryptophan and three lysine amino acids, then its purity was determined by reverse-phase high-performance liquid chromatography. The peptide was confirmed using mass spectrometry and electrospray ionization mass spectrometry. Silver nanoparticles conjugate with Tryasine was synthesized by adding Tryasine-silver nitrate solution in the presence of the reducing agent sodium borohydride. The presence of Tryasine-silver nanoparticles was indicated by the yellow-brown color and was further confirmed through ultraviolet-visible spectrophotometry. The minimum inhibitory and minimum bactericidal concentrations for Tryasine nanoparticles were determined against *Staphylococcus aureus*, *Escherichia coli*, methicillin resistant *Staphylococcus aureus*, and ESBL *Escherichia coli* using the microdilution method. Toxicity for nanoparticles conjugated with Tryasine was determined using erythrocyte hemolytic assay. *Results:* Tryasine alone was effective (MIC around 100 and 200 μM) against standard and resistant strains of bacteria used. However, Tryasine-silver nanoparticles were more effective with MICs ranging from 30 to 100 μM depending on the bacterial strain used. Tryasine-silver nanoparticles at concentration of 100 μM only caused 1% hemolysis on human erythrocytes after 30 min of incubation. *Conclusions:* The findings indicate that Tryasine-silver nanoparticles had good antibacterial activity against pathogenic strains of Gram-positive and Gram-negative bacteria. Additionally, the conjugate showed low hemolytic activity and cytotoxicity. Therefore, conjugation of Tryasine with silver nanoparticles is a promising treatment candidate for bacterial infection with low toxicity.

## 1. Introduction

Antibiotic resistance is one of the most serious problems that the world faces and it is on the rise around the world, posing a hazard to the global population and causing alarm among health authorities and governments [1]. The indiscriminate use of conventional antibiotics to treat a variety of ailments is thought to be a key contributor to the problem. The rise of antibiotic-resistant bacteria has resulted in a dramatic reduction in the number of antibiotics developed for clinical use [2]. Recent decades have witnessed an upsurge in the number of MDR bacteria with few reported to display resistance against all clinically available antimicrobials, ushering the possibility of humans entering the post-antibiotic era and consequently threatening the lives of millions of people around the globe [3]. Plasmid-mediated antibiotic resistance or bacterial chromosome-maintained resistance are both possible. Antibiotic hydrolysis, which is mediated by the bacterial enzyme beta-lactamase, is the most significant mechanism of resistance to penicillins and cephalosporins. Through exposure to beta-lactam medications, the expression of chromosomal beta-lactamase can be stimulated or steadily suppressed. The creation of new antibiotics that are resistant to beta-lactamase attack and the co-administration of beta-lactamase inhibitors with beta-lactam medications are two strategies for overcoming beta-lactam antibiotic resistance. Penicillin-binding protein, an antibiotic target protein, is altered to cause resistance to methicillin, which is stable to Gram-positive beta-lactamase 2. For the other classes of antibiotics, such as trimethoprim, sulfonamides, aminoglycosides, chloramphenicol, and quinolone drugs, the main resistance mechanisms are the production of antibiotic-modifying enzymes and the synthesis of antibiotic-insensitive bacterial targets. For several classes of antibiotics, including beta-lactam medications, aminoglycosides, chloramphenicol, and quinolones, decreased antibiotic penetration is also a resistance mechanism [4].

Therefore, there is an urgent need to find new alternatives for antibiotics or developed strategies to improve the efficacy of the existing ones. One new and promising alternative to traditional antibiotics is antimicrobial peptide (AMP) [5]. AMP is a small molecule (less than 10 kDa) with a variety of sizes and amino acid arrangements. AMPs have positive charges ranging from +3 to +9, as well as more than 30% hydrophobic residues that are amphipathic. When AMPs come into contact with plasma membranes, their amphipathic properties allow them to generate pores in the target membranes, allowing intracellular cell leakage and cell death to occur. Another important target for AMPs is DNA, where they inhibit DNA replication and transcription after crossing the bacterial cytoplasm without damaging the cell membrane [6]. Therefore, these AMPs may offer a substitute for antibiotics or they may work in tandem with antibiotics to combat different infections [7]. Despite the benefits of AMPs as an alternative to conventional antibiotics, a number of issues have hampered their clinical application. These issues arise as a result of the low blood stability caused by lipoproteins and negative charge albumins that they interact with [8]. More importantly, AMPs lack selectivity, resulting in undesired interactions with host cells and a high level of erythrocyte toxicity [9]. Due to these major issues, new research approaches focusing on the design of novel sub-families of AMPs known as ultra-short antimicrobial peptides (USAMPs), which include three to ten amino acids, have been developed. These USAMPs have numerous structural and economic advantages over traditional AMPs, and hence potentially play a key role in overcoming traditional AMP limitations [10]. Furthermore, USAMPs have several advantages for development over AMPs, due to their structural properties and limited number of amino acids, including low cost of production, reduced mammalian cell toxicity, and increased potency. This would eventually provide the scientific community with a viable option of molecules for antimicrobial development. Nanotechnology is a rapidly evolving field with numerous applications in medication research and design. Simultaneously, silver has been employed as an antibacterial and antiseptic substance with little side effects. Silver nanoparticles (AgNPs) are found to be broad-spectrum with good antibacterial, antifungal, and antiviral activity. The mode of action of AgNPs is thought to be through passing the bacterial cell walls, altering the structure of cell membranes and potentially causing cell death by releasing silver ions which interact with the thiol group [11]. As a result, we present the design and characterization of an hexa conjugated ultra-short antimicrobial peptide (Tryasine) composed of alternating W: tryptophan subunits and Lysine: K and to further improve the hydrophobic character of the ultra-short peptide, the hexa was conjugated to ferulic acid. The resultant Tryasine peptide was additionally coupled with AgNPs to boost its efficacy against resistant and standard potentially pathogenic Gram- positive and Gram-negative bacteria.

## 2. Results and Discussion

Infections produced by drug-resistant bacteria are a severe and growing worldwide health concern. As a result, major efforts are conducted in the development of new products [12].

Despite these efforts, a rising number of multidrug-resistant bacteria are reported on a regular basis, including methicillin resistant *S. aureus* (MRSA), extended-spectrum beta-lactamase *Escherichia coli* (ESBL *E. coli*) [13].

In this work, a novel peptide Tryasine and conjugate of this peptide with silver nanoparticles (Tryasine-AgNPs) was studied against important pathogenic bacteria, including *S. aureus*, *E. coli*, MRSA, and ESBL *E. coli*.

**Figure 1:**
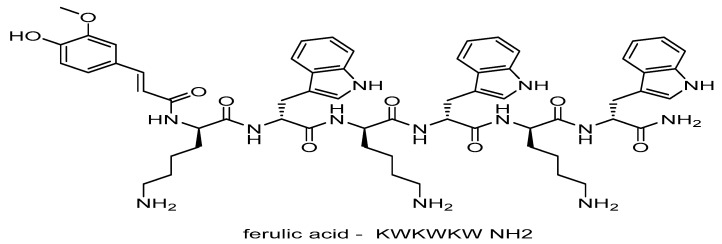
The overall structure of Tryasine, which has a net positive charge of +2 and a molecular weight of 1136.34 g/mol.

**Figure 2:**
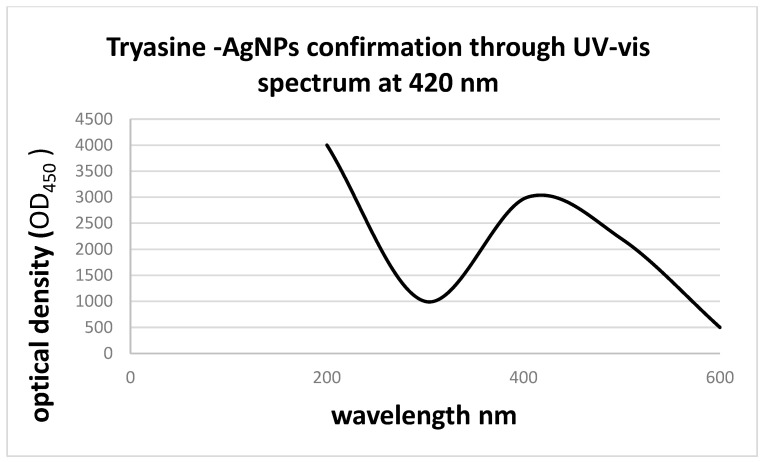
Absorption had shown silver nanoparticles surface plasmon resonance at 420 nm.

**Figure 3:**
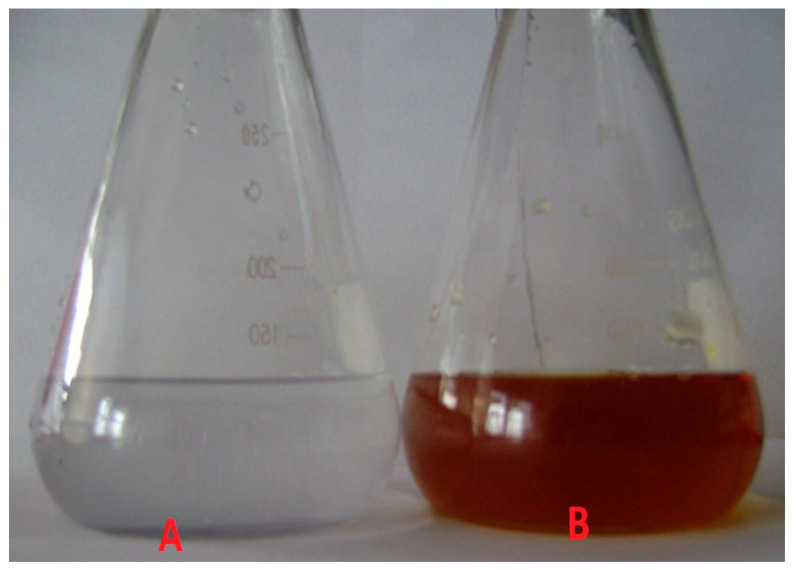
The color change before and after the reaction. (**A**) The color before, (**B**) the color after the reaction.

Tryasine was synthesized and confirmed using mass spectrometry and electrospray ionization mass spectrometry (Figure 1). Then, the peptide was conjugated with AgNPs. Figure 2 shows Tryasine-AgNPs confirmation through UV-Vis spectrum at 420 nm, the color change after the reaction was also shown in Figure 3. Moreover, in Table 1, the result of zeta potential of the nanoparticles was shown.

**Figure 4:**
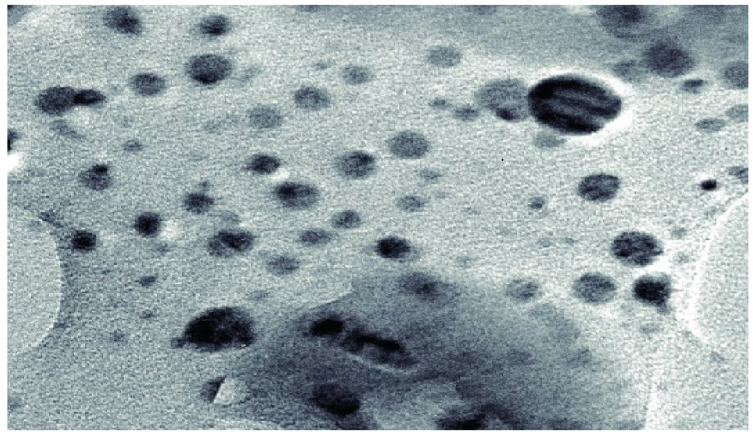
High-resolution transmission electron microscopy image of silver nanoparticles nucleated on GPG-AG3 after 3 days. The dark spots are silver nanoparticles.

To confirm the conjugation, Figure 4 shows a transmission electron microscopy image of silver nanoparticles after the combination.

Table 2 shows that the peptide Tryasine is effective against all standard and resistant Gram-positive and Gram-negative bacterial strains used, and thus has the potential for use as a therapeutic alternative to conventional antibiotics. The antibacterial activity was better against the standard strains of *E. coli* and *S. aureus* with an MIC/MBC of 70 and 80 µg mL^−1^, respectively. The peptide was also effective against MRSA and ESBL *E. coli* (MIC/MBC = 180 and 188 µg mL^−1^, respectively), which indicates a good potential for use on infections with resistant strains when other medications might fail. However, when compared with the other AMPs, the MIC values were slightly greater, which can be explained by the Tryasine net charge. The ideal net charge should be +3 for antibacterial activity; however, our peptide had a net charge of +2, which might account for the lower antibacterial activity [14].

Nanotechnology has a large amount of potential, especially for diagnostics and drug delivery. Medication delivery methods based on nanomaterials have the potential to improve drug pharmacokinetics and pharmacodynamics [13]. Various drug-binding NPs have been created to eradicate drug-resistant bacterial infections, since the smaller nanoparticle size yields a bigger surface area for maximal drug delivery and availability [14]. In an attempt to improve Tryasine antibacterial activity, the peptide was conjugated with silver nanoparticles. The conjugation of Tryasine with silver nanoparticles resulted in reduction in the peptide’s MIC value in this investigation, sometimes to around 50% (Table 1). The drop in the MIC could be due to the fact that Tryasine pours into the outer membrane of the bacteria cell wall, increasing its permeability, and therefore increasing the antibiotic impact of silver nanoparticles [15].

To compare the efficacy of our conjugate, we studied the effect of conventional antibiotics against the same type of bacteria. Additionally, the results of MIC and MBC values were shown in Table 3 and Table 4, indicating that our peptide has good efficacy compared with traditional antibiotics.

The Tryasine-AgNPs proved to be an effective antibacterial agent with bactericidal mode of action, as indicated by similar MICs and MBCs. To explore its toxicity, the effect of this conjugate to damage mammalian erythrocytes, in particular, was assessed using the standard erythrocytes hemolysis assay [16]. The erythrocytes were challenged with different concentrations of the conjugate ranging from 5–100 μg mL^−1^. The obtained results revealed that the conjugate caused only 1% hemolysis after 30 min of incubation with human erythrocytes at a concentration of 100 μg mL^−1^ (Table 5). Therefore, the hemolytic assay confirmed that the conjugate exhibits negligible hemolytic activity.

**Figure 5:**
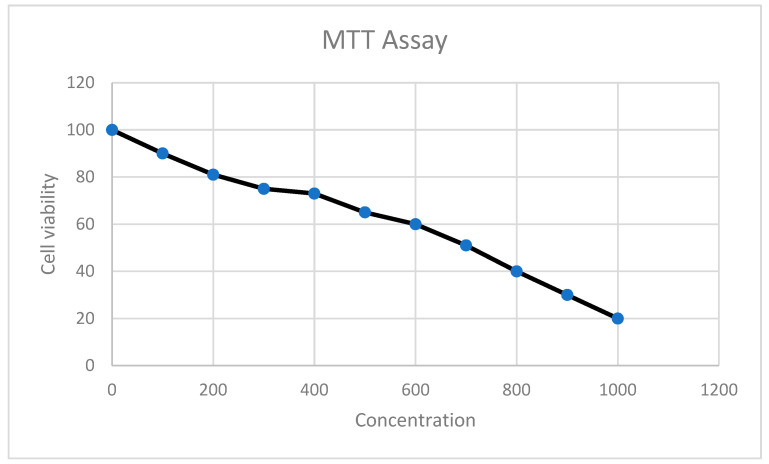
MTT assay results after the treatment with the conjugate.

The results of the cytotoxicity assay revealed that the conjugate has an IC50 value of 185.6 g/mL (Figure 5).

It has been proven that antibacterial peptides are considered promising medication candidates [17]; however, worries about cell toxicity, metabolic stability, and expensive manufacturing costs have delayed their development [18]. Our peptide displays good antibacterial efficacy against a number of typical pathogenic and resistant pathogens, including MRSA and ESBL *E. coli*, with a bactericidal mechanism of action, as reported in this study. Additionally, Tryasine-AgNPs conjugate showed strong activity against the type of bacteria we tested, while exhibiting low hemolytic activity and cytotoxicity. Therefore, the conjugation method with AgNPs showed significant advantages in terms of peptide antibacterial activity and toxicity.

## 3. Materials and Methods

### 3.1. Bacterial Cultures

*Staphlococcus aureus* (ATCC 29213), *Escherichia coli* (ATCC 25922), methicillin resistant *S. aureus* (MRSA) (ATCC BAA-41), and ESBL *E. coli* (ATCC BAA-3054), were obtained from the American Type Tissue Culture Collection (ATCC, Manassas, VA, USA) and were used in the study.

### 3.2. Design and Synthesis of Tryasine

Three subunits of tryptophan (w) and three lysine (K) amino acids were used to create Tryasine. Thereafter, ferulic acid was added to conjugate the peptide. The solid-phase Fmoc chemical was used to develop Tryasine. Reverse-phase high-performance liquid chromatography (RPHPLC) was used to assess the purity of Tryasine, which was further confirmed using mass spectrometry and electrospray ionization mass spectrometry (ESIMS LCMS-8060NX) [19].

#### 3.2.1. Nanoparticles (NPs) Characterization

The hydrodynamic radius of NPs was measured using dynamic light scattering (DLS). At 20 °C, 1 mL (0.5 mg/mL) of each sample was added to disposable polystyrene cuvettes. The developed NPs’ zeta potential (ZP) was measured using a zetasizer ZS (Malvern, UK) at 25 °C in 10 mM phosphate buffer saline PBS, pH 7.4. The samples (0.5 mg/mL) were filtered through a 0.45 m filter unit prior to injection into folded capillary cells [20].

#### 3.2.2. Field Emission Scanning Electron Microscopy

Samples were dropped onto carbon tape and remained to dry at room temperature. Subsequently, samples were visualized using a field emission scanning microscope (6340F; Jeol Ltd., Tokyo, Japan), using an accelerating voltage of 5 kV and an emission current of 12 mA.

### 3.3. Minimum Inhibitory Concentrations (MICs) and Minimum Bactericidal Concentrations (MBCs) Determination of Tryasine

The MIC and MBC of Tryasine was assessed using sterile 96-well plates, in accordance with the Clinical and Laboratory Standards Institute [21]. The bacteria were grown on Muller Hinton Broth (MHB), then diluted to 10^6^ CFUmL^−1^ in the same medium. Several dilutions of Tryasine with final concentrations ranging from 0.5 to 100 M were prepared. An aliquot of 50 µL of each solution were poured in the wells of the 96-well plates, to which 50 µL of diluted bacterial suspension were added. Each peptide concentration test was repeated in three consecutive wells. The plates were then incubated for 18 h at 37 °C. The bacterial growth was quantified using an ELISA-OD plate reader at 570 nm. A column in the plate was used as positive control, where the wells containing 50 µL MHB were inoculated with 50 µL bacterial suspension without antimicrobial agents. Another column was used for negative control, where 100 µL of MHB was added alone. MBCs were determined by taking 10 µL from clear wells and cloudy positive control wells, which were seeded on sterile agar medium and incubated for 24 h at 37 °C. The concentration that causes 0.1% live cells was considered as the MBC value.

### 3.4. Synthesis of AgNPs Conjugate with Tryasine (Tryasine-AgNPs)

An aliquot of 5 mL (0.1 mM) of Tryasine solution was combined with 5 mL (0.1 mM) silver nitrate solution and agitated for 10 min. An amount of 20 mL of 5 mM sodium borohydride solution (NaBH_4_) was added to the mixture. A reducing agent (sodium borohydride solution (NaBH_4_) was added to the solution to cause silver ion reduction and then the creation of Tryasine-AgNPs, which was indicated by the color change from clear to yellow-brown. The nanoparticles (NPs) were centrifuged at 12,000× *g* for 1 h, after which the supernatant layer was collected and freeze-dried. The yield was estimated as a percentage of the active component content in 100 mg of dry nanoparticles [22].

### 3.5. Minimum Inhibitory Concentrations (MICs) and Minimum Bactericidal Concentrations (MBCs) Determination of Tryasine-AgNPs

Bacterial cells were cultivated overnight in MHB, and then diluted in the same medium to yield a concentration of 10^6^ CFU mL^−1^ prior to use. Different dilutions with sterile distilled water were prepared with Tryasine-AgNPs to yield final concentrations ranging from 0.5 to 100 µg mL^−1^.

An aliquot of 50 μL of each concentration and 50 μL of diluted bacterial solution were placed in each well in 96-well plates. Three replicates of each peptide concentration were performed each time. Plates were incubated for 24 h at 37 °C. Bacterial growth was determined by measuring the OD at 570 nm using an enzyme-linked immunosorbent assay (ELISA) for plate stability and the MIC was determined as the minimum concentration that inhibited growth (turbidity). Each plate contained a positive control (50 μL of bacterial suspension plus 50 μL of MHB without antibacterial drugs) and a negative control of 200 μL MHB). Each experiment was repeated three times. MBCs were determined by taking 10 μL from clear negative wells and turbid positive control wells, after which the aliquots were streaked on sterile labeled nutrient media [23].

The fold change in MIC/MBC as a result of combination was calculated as follows:

(1) $$\mathrm{The}\;\mathrm{fold}\;\mathrm{change}\;\mathrm{in}\;\mathrm{MIC}\;=\frac{\mathrm{MIC}/\mathrm{MBC}\;\mathrm{for}\;\mathrm{TRYASINE}\;\mathrm{alone}\;}{\mathrm{MIC}/\mathrm{MBC}\;\mathrm{for}\;\mathrm{TRYASINE}-\mathrm{AgNPs}}\;\times 100\%$$

### 3.6. Erythrocyte Hemolytic Assay

A conventional hemolytic experiment was used to test the capacity of Tryasine conjugation with silver nanoparticles to destroy mammalian erythrocytes. An aliquot of 2 mL of human blood was transferred into a 50-mL centrifuge tube at 3000× *g* for 5 min. The supernatant was then collected, and the cell pellet was suspended in 48 mL of phosphate-buffered saline and centrifuged at 3000× *g* for 5 min three times. The cells were suspended in sterile tubes containing 50 mL of PBS to obtain a final concentration of 4% erythrocytes. An amount of 1 mL of each peptide concentration was added to 1 mL of erythrocyte suspension (4%). Positive controls were prepared by diluting 1 mL of the erythrocyte solution with 5 μL of triton X-100. Negative controls were prepared by combining 1 mL of erythrocyte suspension with 1 mL of PBS. The suspension was incubated at 37 °C for 1 h. The mixture was gently mixed and 1 mL of each sample was aspirated into sterile Eppendorf tubes, then centrifuged at 3000× *g* for 5 min. An amount of 100 μL of the mixture were taken from each Eppendorf and placed in a 96-well plate [24]. At a wavelength of 570 nm, the absorbance was measured. The following formula was used to compute the percentage of hemolysis:

(2) $$\%\;\mathrm{Hemolysis}=\frac{\left(A\;-\;\mathrm{AO}\right)\;}{\left(\mathrm{AX}\;-\mathrm{AO}\right)}\;\times \;100$$

where A is optical density 450 with the peptide solution, AO is optical density 450 of the blank, and AX is optical density 450 of control (0.1% triton X-100).

## 4. MTT Cell Proliferation Assay

The cell line used in this study was the mammalian Vero cell line, which was purchased commercially from ATCC (ATCC CCL81). Yellow tetrazolium (3-(4, 5-dimethylthiazolyl-2)-2, 5-diphenyltetrazolium bromide) MTT is reduced to purple formazan inside the cell by reductase enzymes. As a result, only metabolically active cells can catalyze this reaction and generate the purple formazan crystals. Although these purple crystals are insoluble in water, they can be dissolved in dimethyl sulfoxide (DMSO). The generated color of these crystals can be measured spectrophotometrically at 550 nm wavelength. Cells were seeded in 5 × 10^3^ cells per well in a flat-bottomed 96-well plate for the MTT assay, and the plates were incubated for 18–24 h at 37 °C supplemented by 5% CO_2_ for attachment on the bottom of the plates. The following day, different concentrations of Tryasine-AgNPs were suspended in RPMI as the dissolving media and added to the cells in the plates (2, 4, 6, 8, and 10 mg/mL), loaded with (200, 400, 600, 800, and 1000 g/mL) of Tryasine-AgNPs, respectively). As a control, the untreated medium is used. The plates were incubated for 24 h at 37 °C with 5% CO_2_ added. After 24 h, 20 μL of the MTT solution (2.5 mg/mL) was added to each well, and the plates were incubated for 2–5 h at 37 °C supplemented by 5% CO_2_. The well content was removed after this incubation period (ensure that all of the solution in the wells is removed). Each well received 100 μL of DMSO, which was thoroughly mixed by pipetting to dissolve the formazan crystals at the bottom of the wells until a clear purple color was obtained. Then, the plates were placed on an absorbance microplate reader (BioTek, Winooski, VT, USA) and the absorbance at 550 nm was measured [20].

## 5. Conclusions

In this study, we report on the design and antimicrobial characterization of a novel conjugate of Tryasine with silver nanoparticles, which showed promising activities against clinically important resistant Gram-positive and Gram-negative bacteria with negligible hemolytic activities. Therefore, the conjugation of Tryasine with silver nanoparticles can be a promising treatment candidate for bacterial infection with low toxicity. The peptide-silver nanoparticles conjugate has been shown to have a significantly higher stability and activity compared with the nanoparticle or peptide alone. This conjugate could be the future solution in overcoming the increase of antimicrobial resistance, which is due to its unique characteristic compared with traditional antibiotics.

## Figures and Tables

**Table 1:** The characterization of AgNPs and Tryasine-AgNPs: Surface charge (zeta potential) and average size diameter.

Formulation	Zeta Potential (mV ± SD)	Size (nm ± SD)	PdI ^a^
Ag-NP	+40.8 ± 2.55	102.27 ± 0.5	0.255
Tryasine-AgNPs	+31.2 ± 2.1	118.23 ± 1.01	0.272

^a^ Polydispersity index of the diameter distribution peak.

**Table 2:** MIC and MBC of Tryasine and Tryasine-AgNPs conjugate on standard and resistant bacterial strains.

Bacterial Strains	MIC µg mL^−1^			MBC µg mL^−1^		
	AgNPs Alone	Tryasine Alone	Tryasine-AgNPs	Tryasine Alone	Tryasine-AgNPs	Fold Change in MIC/MBC
*S. aureus* (ATCC 29215)	120	80	30	80	30	37%
Methicillin Resistant *S. aureus* (MRSA) (ATCC BAA-41)	230	180	90	180	90	50%
*E. coli* (ATCC 25922)	140	70	28	70	28	40%
ESBL *E. coli* (ATCC BAA-3054)	220	188	78	188	78	50%

**Table 3:** Minimum inhibitory concentrations in μM of the eight antibiotics against the tested bacterial strains.

Antibiotics	*S. aureus* (ATCC 29215)	MRSA (ATCC BAA-41)	*E. coli* (ATCC 25922)	ESBL *E. coli* (BAA-3054)
**Levofloxacin**	0.5	10	2	12
**Chloramphenicol**	20	25	80	150
**Rifampicin**	0.025	0.005	15	50
**Amoxicillin**	5	40	25	200
**Clarithromycin**	0.5	125	125	125
**Doxycycline**	2	10	1.5	16
**Vancomycin**	0.5	2	200	250
**cefixime**	4	30	6	80

**Table 4:** Minimum bactericidal concentrations in μM of the antibiotics against the tested bacterial strains.

Antibiotics	*S. aureus* (ATCC 29215)	MRSA (ATCC BAA-41)	*E. coli* (ATCC 25922)	ESBL *E. coli* (BAA-3054)
**Levofloxacin**	0.5	10	2	12
**Chloramphenicol**	30	40	100	200
**Rifampicin**	0.025	0.005	15	50
**Amoxicillin**	5	40	25	250
**Clarithromycin**	1.5	150	150	200
**Doxycycline**	10	20	15	25
**Vancomycin**	0.5	2	150	200
**cefixime**	4	30	6	80

**Table 5:** The in vitro hemolysis activity of Tryasine-AgNPs in human erythrocytes.

Concentration μg mL^−1^	Hemolysis of AgNPs %	Hemolysis of Tryasine %	Hemolysis of Tryasine-AgNPs %
5	80	0	0
10	85	0	0
20	87	0	0
40	95	0	0
60	100	1	0
80	100	1	0
100	100	2	1

## Data Availability

The data are available upon request by email from the authors.
